# Hemolytic Potential of Tafenoquine in Female Volunteers Heterozygous for Glucose-6-Phosphate Dehydrogenase (G6PD) Deficiency (*G6PD Mahidol* Variant) versus G6PD-Normal Volunteers

**DOI:** 10.4269/ajtmh.16-0779

**Published:** 2017-07-24

**Authors:** Ronnatrai Rueangweerayut, Germana Bancone, Emma J. Harrell, Andrew P. Beelen, Supornchai Kongpatanakul, Jörg J. Möhrle, Vicki Rousell, Khadeeja Mohamed, Ammar Qureshi, Sushma Narayan, Nushara Yubon, Ann Miller, François H. Nosten, Lucio Luzzatto, Stephan Duparc, Jörg-Peter Kleim, Justin A. Green

**Affiliations:** 1 Department of Internal Medicine, Mae Sot General Hospital, Mae Sot, Thailand;; 2 Shoklo Malaria Research Unit, Mahidol–Oxford Tropical Medicine Research Unit, Faculty of Tropical Medicine, Mahidol University, Mae Sot, Thailand;; 3 GlaxoSmithKline Research and Development Ltd., Uxbridge, United Kingdom;; 4 GlaxoSmithKline, Research Triangle Park, North Carolina;; 5 Faculty of Medicine, Siriraj Hospital, Mahidol University, Bangkok, Thailand;; 6 Medicines for Malaria Venture, Geneva, Switzerland;; 7 GlaxoSmithKline, Bangkok, Thailand;; 8 GlaxoSmithKline, King of Prussia, Pennsylvania;; 9 Istituto Toscano Tumori, Florence, Italy;; 10 Department of Hematology and Blood Transfusion, Muhimbili University of Health and Allied Sciences, Dar-es-Salaam, Tanzania

## Abstract

Tafenoquine is an 8-aminoquinoline under investigation for the prevention of relapse in *Plasmodium vivax* malaria. This open-label, dose-escalation study assessed quantitatively the hemolytic risk with tafenoquine in female healthy volunteers heterozygous for the *Mahidol*^*487A*^ glucose-6-phosphate dehydrogenase (G6PD)-deficient variant versus G6PD-normal females, and with reference to primaquine. Six G6PD-heterozygous subjects (G6PD enzyme activity 40–60% of normal) and six G6PD-normal subjects per treatment group received single-dose tafenoquine (100, 200, or 300 mg) or primaquine (15 mg × 14 days). All participants had pretreatment hemoglobin levels ≥ 12.0 g/dL. Tafenoquine dose escalation stopped when hemoglobin decreased by ≥ 2.5 g/dL (or hematocrit decline ≥ 7.5%) versus pretreatment values in ≥ 3/6 subjects. A dose–response was evident in G6PD-heterozygous subjects (*N* = 15) receiving tafenoquine for the maximum decrease in hemoglobin versus pretreatment values. Hemoglobin declines were similar for tafenoquine 300 mg (−2.65 to −2.95 g/dL [*N* = 3]) and primaquine (−1.25 to −3.0 g/dL [*N* = 5]). Two further cohorts of G6PD-heterozygous subjects with G6PD enzyme levels 61–80% (*N* = 2) and > 80% (*N* = 5) of the site median normal received tafenoquine 200 mg; hemolysis was less pronounced at higher G6PD enzyme activities. Tafenoquine hemolytic potential was dose dependent, and hemolysis was greater in G6PD-heterozygous females with lower G6PD enzyme activity levels. Single-dose tafenoquine 300 mg did not appear to increase the severity of hemolysis versus primaquine 15 mg × 14 days.

## INTRODUCTION

An estimated 132–391 million *Plasmodium vivax* malaria cases occur annually, mostly in India, south and southeast Asia, South America, and the Horn of Africa.^1^ *Plasmodium vivax* hypnozoites lie dormant in the host liver; their reactivation causing clinical relapses, with substantial health and economic consequences. For more than 60 years, primaquine (plus a blood schizonticide) has been the only drug available for the prevention of relapse in *P. vivax* malaria. Although primaquine remains efficacious in preventing *P. vivax* relapse, the 7- to 14-day dosing schedule diminishes treatment effectiveness as poor adherence is associated with clinical failure.^2^ In 2012, the World Health Organization collected data on the number of primaquine treatment courses reported by 24 national programs and, based on the estimated number of *P. vivax* cases in public facilities for each country, concluded that only 10% of *P. vivax* cases receive primaquine treatment at therapeutic doses.^3^ Clearly, there is an unmet need for a therapy that is convenient for patients and appropriate for use in a public health context.^4^

Observations of primaquine-induced hemolysis led ultimately to the identification of inherited glucose-6-phosphate dehydrogenase (G6PD) deficiency.^5^ G6PD catalyzes the first step in the pentose phosphate pathway, protecting against oxidative stress by generating reduced nicotinamide adenine dinucleotide phosphate (NADPH) for reduction cycling of glutathione. The erythrocyte, lacking alternate pathways, is uniquely dependent upon G6PD for NADPH production. Primaquine metabolism produces reactive oxygen species (ROS), which rapidly deplete glutathione in G6PD-deficient erythrocytes: the crucial role of glutathione in ROS-related hemolysis is demonstrated by the fact that very rare patients with glutathione reductase deficiency can suffer attacks of acute hemolytic anemia (AHA) very similar to those of G6PD-deficient persons.^6^ Glutathione depletion is followed by damage to hemoglobin and presumably to other intraerythrocytic and membrane proteins. The Heinz bodies that can be observed, early in the course of a primaquine-induced hemolytic episode, on supravital staining of peripheral blood are intracellular precipitates of denatured hemoglobin. Pitting of Heinz bodies by the spleen produces “bite cells”; whereas cross-linking of membrane proteins produces “hemi-ghosts” in which the hemoglobin is only present in one part of the red cells.^7^ Thus, the red cell abnormalities seen on a peripheral blood smear, which include numerous spherocytes, are rather spectacular and virtually pathognomonic of oxidative hemolysis. There is evidence that intravascular hemolysis (hence the hemoglobinuria) and extravascular hemolysis (hence the hyperbilirubinemia) coexist in every case (as best studied in favism).^8^ It is likely that the most severely damaged red cells undergo intravascular hemolysis, whereas others are removed by macrophages in the spleen and elsewhere, thus giving rise to extravascular hemolysis.

The overall median frequency of G6PD deficiency alleles in malaria endemic regions is estimated at 8.0%^9^ though it can reach > 30% in some populations.^9^ Except for some rare severely deficient variants, most individuals with G6PD deficiency are asymptomatic unless exposed to oxidative stress. G6PD deficiency is geographically associated with regions of malaria, either current or historical, and is thought to be associated with some protection from lethal malaria.^10–12^ The G6PD gene is on the X-chromosome, consequently, G6PD-deficient hemizygous males and homozygous females have only G6PD-deficient red cells and are at greatest risk of severe AHA.^13^ Lyonization in females heterozygous for G6PD-deficient variants results in highly variable proportions of G6PD-deficient and G6PD-normal red cells, the phenotype ranging from that of hemizygous deficient males to that of G6PD-normal individuals.

Tafenoquine, coadministered with a 3-day chloroquine course, is in Phase III development as a single-dose treatment for the prevention of *P. vivax* relapse.^14–17^ A recent Phase IIb dose-ranging study identified tafenoquine 300 mg as the minimally effective dose (when given with chloroquine): relapse-free efficacy at 6-months was 89.2% (95% confidence interval 77–95) with tafenoquine 300 mg and 77.3% (63–87) with primaquine 15 mg for 14 days plus chloroquine.^4,17^ Tafenoquine, like primaquine, is an 8-aminoquinoline derivative and also causes hemolysis in G6PD-deficient individuals, including heterozygotes. However, it was unknown whether the longer half-life of tafenoquine versus primaquine would increase the risk and degree of G6PD-associated hemolysis.

Currently, there is no approved method of evaluating the hemolytic potential of new drugs in G6PD-deficient individuals. The risk of severe AHA is greatest in G6PD-deficient hemizygous males or homozygous females; and is considerably less, on average, in heterozygous females.^13^ However, the occurrence and severity of drug-induced AHA in heterozygotes cannot be predicted from the G6PD genotype because it depends on the post-Lyonization phenotype, that is, on the percentage of G6PD-deficient red cells in the blood.^13^ Thus, by selecting G6PD-heterozygotes with sufficiently high G6PD functional enzyme levels for dose–response testing with a potentially hemolytic drug, the risk of severe AHA is minimized, at the same time providing information regarding the relative hemolytic risk associated with different agents.

The primary objective of this study was to evaluate the safety, tolerability, and hemolytic potential of tafenoquine in G6PD-heterozygous female volunteers with *G6PD Mahidol* and moderately decreased G6PD enzyme activity (40–60%), versus G6PD-normal female healthy volunteers, with reference to the hemolytic potential of primaquine.

## MATERIALS AND METHODS

### Design.

The study was conducted in two centers in Thailand (Bangkok and Mae Sot) between July 2, 2009 and April 1, 2013. Tafenoquine hemolytic potential was investigated in healthy female volunteers heterozygous for *G6PD Mahidol* (henceforth referred to as G6PD heterozygous) versus those homozygous for a G6PD-normal genotype (henceforth referred to as G6PD normal). The final protocol, including five amendments, is available from the corresponding author. No formal statistical hypotheses were tested and individual subject data are presented. Sample size was based on feasibility.

This open-label, tafenoquine single-dose, dose-escalation study used a step-wise, risk exposure approach. Each tafenoquine dose level was to be given to a maximum of six G6PD-heterozygous subjects with an enzyme activity range 40–60% of the adjusted site defined median value for G6PD-normal males (median 11.54 IUg/Hb) and six G6PD-normal subjects (G6PD activity ≥ 90% of adjusted site median normal value). The adjusted site median normal value was determined from 36 males by measuring G6PD activity in those with hemoglobin levels ≥ 12 g/dL, after excluding outlying results with G6PD activity ≥ 2 standard deviations of the mean.^18^

Tafenoquine (GlaxoSmithKline, Harlow, United Kingdom) dosing started at 100 mg, with planned dose escalation to 200, 300, 400, and 600 mg. Dose escalation was halted if ≥ 3/6 subjects in any cohort had a dose-limiting toxicity, defined as a hemoglobin decrease of ≥ 2.5 g/dL versus pretreatment values (or hematocrit decline ≥ 7.5%), or any significant signs and symptoms of hemolysis. The maximum tolerated dose was defined as the dose at which < 3/6 G6PD-heterozygous subjects experienced a dose-limiting toxicity. Once determined, the maximum tolerated dose was administered to two further cohorts, each including a maximum of six G6PD-heterozygous subjects, one with G6PD enzyme levels 61–80% and the other with G6PD enzyme levels > 80% of the site median normal value.

Primaquine (Sanofi-Aventis, Bridgewater, NJ), 15 mg once daily for 14 days, was used as an active control in up to six G6PD-heterozygous subjects with an enzyme activity range 40–60% of the site median normal value and six G6PD-normal subjects. Primaquine stopping criteria were the same as for tafenoquine.

### Study participants.

Subjects were female healthy volunteers aged 18–45 years, nonpregnant and nonlactating and with alanine aminotransferase, alkaline phosphatase, and bilirubin ≤ 1.5× the upper limit of normal. Subjects were excluded if they had any clinically significant illness; relevant biological or physical abnormality; symptoms of severe vomiting; a history of hemoglobinopathy or methemoglobinemia > 3%; treatment with an investigational drug within 30 days or five half-lives; blood donation of > 0.5 L within the previous 56 days; pretreatment hemoglobin < 12.0 g/dL or hematocrit < 36%. G6PD heterozygous subjects had *G6PD Mahidol* identified by genotyping and G6PD enzyme activity within the defined range for the cohort.

### Procedures.

Blood samples (10 mL) were collected for pharmacogenomics at screening. Genotyping by polymerase chain reaction restriction fragment length polymorphism was performed using standard methods.^19^ Initially, manual cytochemical staining was used to define enzyme activity.^20^ However, after recruitment of the first cohort (100 mg tafenoquine) was completed, it became evident that cytochemical analysis was technically challenging and not as reliable as enzyme activity measurements in this setting. Thus, for all subsequent cohorts a quantitative enzyme assay was performed on lysed red cells.^21^ This was more reproducible and, coupled with definition of a local median in normal males, allowed entry to be based on the G6PD activity as a percentage of the site median normal value. Briefly, G6PD activity was assessed using a standard protocol,^21^ without correction for 6-phosphogluconate dehydrogenase. Samples were depleted of white blood cells using CF11 columns,^22^ and hemolysates were obtained by freeze-thaw overnight at −80°C; analysis was performed at 30°C within 30 minutes after thawing using reagents prepared in the laboratory. Hemoglobin concentration was assessed using Drabkin’s method on the same hemolysates.

G6PD-heterozygous subjects were admitted to the unit from day –1 to 14. G6PD-normal subjects were hospitalized from day 1 to 2, returning daily as outpatients until day 14. Subsequent out-patient follow-up visits were at days 21, 28, and 56. Safety assessments included adverse event monitoring, vital signs, and 12-lead electrocardiograms, clinical biochemistry, hematology (including methemoglobin determined by oximetry) and urinalysis.

The hemoglobin concentration of each sample was measured using locally available semiautomated machines. Reticulocytes were visualized by staining with supravital stain (methylene blue) based on triplicate slide readings. Clinical biochemistry tests and normal values are shown in Supplemental Table 1. Serial blood samples were taken for pharmacokinetic assessments. Plasma samples were analyzed by GlaxoSmithKline using validated methods based on protein precipitation, followed by high-performance liquid chromatography tandem mass spectrometry analysis.^23^ Quality control samples were processed against calibration standards and all analytical runs met predefined acceptance criteria.

### Outcomes.

The primary endpoint was maximum absolute decrease in hemoglobin or hematocrit from pretreatment values up to and including day 14 following treatment in G6PD-heterozygous versus G6PD-normal subjects. Pretreatment levels were calculated as the mean hemoglobin or hematocrit of day −1 and day 1 (prior to drug treatment). The maximum decrease in hemoglobin or hematocrit for tafenoquine relative to primaquine in G6PD-deficient and G6PD-normal subjects was a secondary endpoint. Subjects who received at least one dose of study medication were included in the analysis.

Additional outcomes were findings from hematologic laboratory tests. Laboratory findings noted as hemolytic events were dose-limiting toxicity (defined earlier); hemoglobin decline of 1.5 g/dL; hematocrit decline of 4.5%; haptoglobin ≤ 0.25 μg/dL; indirect bilirubin increase of > 50% versus pretreatment; or reticulocytes ≥ 4%. Tafenoquine and primaquine pharmacokinetic parameters, safety, and tolerability were also evaluated.

### Ethical approval.

The study was conducted in accordance with Good Clinical Practice, applicable country-specific requirements, and the Declaration of Helsinki 2008. The participating institutions’ ethics committees approved the protocol. All subjects provided written informed consent prior to study participation.

## RESULTS

### Subjects and study flow.

**Figure 1.**
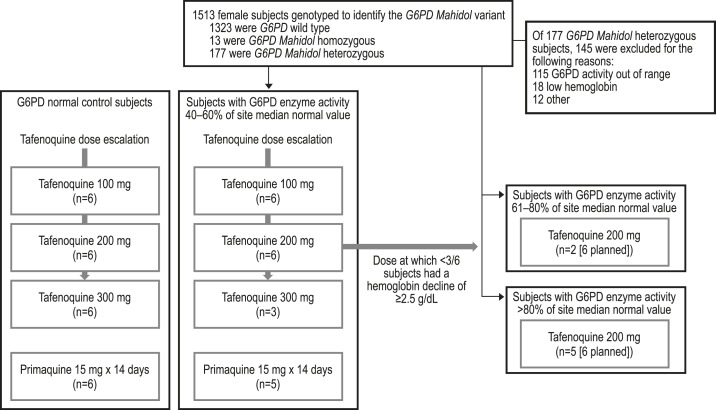
Study flow.

The study included 51 subjects (Figure 1). One subject in the primaquine group withdrew consent on day 6 for personal reasons. Recruitment of two additional cohorts to receive the maximum tolerated dose was suspended during investigation of a potential genotoxic tafenoquine metabolite identified in urine from this study (100 mg cohort) that triggered an in silico alert, but after investigation with an Ames test, it was found not to be genotoxic in vitro and the study recommenced. The study was concluded at that point as 7/12 planned G6PD-heterozygous subjects had been recruited and this was felt to be sufficient to evaluate tafenoquine hemolytic potential. Table 1 shows subject pretreatment characteristics; all were of Asian heritage. In the tafenoquine 100 mg group, some subjects had G6PD enzyme activity outside the defined range because cytochemical staining (rather than G6PD activity) had been used at screening (see earlier).

**Table 1:** Demographic and pre-treatment clinical characteristics of study participants

Characteristic	TQ 100 mg		TQ 200 mg		TQ 300 mg		PQ 15 mg × 14 days		TQ 200 mg	
	Normal (*N* = 6)	Het. (*N* = 6)	Normal (*N* = 6)	Het. (*N* = 6)	Normal (*N* = 6)	Het. (*N* = 3)	Normal (*N* = 6)	Het. (*N* = 5)	Het. A (*N* = 2)	Het. B (*N* = 5)
Mean age, years (SD) (range)	28.7 (7.8) (22–42)	24.3 (5.4) (19–33)	23.3 (3.1) (19–28)	27.5 (9.2) (18–39)	31.8 (4.5) (24–36)	20.7 (3.1) (18–24)	27.0 (6.2) (20–35)	26.6 (8.1) (20–40)	28.5 (13.4) (19–38)	27.2 (9.5) (19–42)
Mean hemoglobin, g/dL (SD) (range)	12.7 (0.8) (12.2–14.3)	12.8 (0.4) (12.3–13.4)	13.3 (0.9) (12.3–14.6)	12.7 (0.2) (12.5–13.1)	13.2 (1.0) (12.2–15.0)	12.4 (0.2) (12.3–12.7)	12.6 (0.5) (12.2–13.3)	12.8 (0.6) (12.0–13.7)	13.1 (1.4) [12.1–14.1)	12.6 (0.5) (12.2–13.4)
Mean hematocrit, % (SD) (range)	38.2 (2.3) (35.7–42.4)	38.6 (1.4) (36.4–40.4)	39.4 (2.8) (36.3–43.4)	38.8 (1.9) (37.0–42.5)	39.8 (2.3) (37.0–43.8)	36.8 (0.9) (35.9–37.7)	36.7 (1.1) (35.6–38.4)	38.3 (1.7) (36.4–40.8)	38.9 (2.4) (37.3–40.6)	37.5 (1.0) (36.5–38.8)
Mean G6PD activity, IU/gHb (SD) (range)	13.2 (7.8) (4.4–23.3)	3.6 (1.5) (2.2–5.8)	12.2 (1.0) (10.9–13.6)	6.2 (0.2) (6.0–6.6)	13.8 (2.9) (11.1–18.8)	5.9 (1.0) (4.7–6.6)	12.5 (0.9) (11.4–13.7)	5.7 (0.7) (5.0–6.7)	7.9 (1.0) (7.2–8.6)	10.2 (1.1) (9.5–12.1)
Mean G6PD activity, % site median normal* (SD) (range)	114.6 (67.4) (38.5–201.9)	31.4 (12.7) (18.9–49.9)	105.4 (8.7) (94.2–118.1)	53.8 (1.8) (51.6–56.8)	119.1 (25.2) (96.0–162.9)	50.7 (8.5) (41.0–56.9)	108.0 (7.4) (98.4–118.4)	49.0 (5.9) (43.6–57.8)	68.6 (8.8) (62.4–74.8)	88.6 (9.1) (82.5–104.8)

G6PD = glucose-6-phosphate dehydrogenase; Het. = G6PD heterozygous 40–60% enzyme activity of site median normal value (19–50% for tafenoquine 100 mg); Het. A = G6PD heterozygous 61–80% enzyme activity of site median normal value; Het. B = G6PD heterozygous > 80% enzyme activity of site median normal value; PQ = primaquine; SD = standard deviation; TQ = tafenoquine.

* Enzyme activity percentage derived from site median normal value of 11.5 IU/gHb.

### Hemolytic potential.

**Figure 2.**
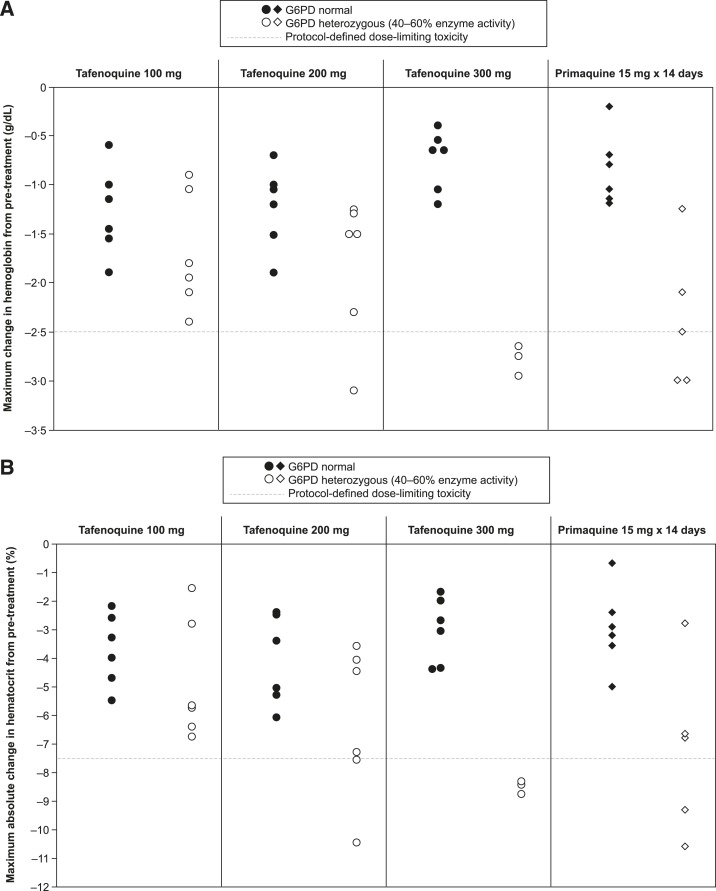
(**A**) Dose–response for the maximum change in hemoglobin from pretreatment values up to and including day 14 following treatment start with single-dose tafenoquine 100–300 mg in glucose-6-phosphate dehydrogenase (G6PD)-deficient heterozygous subjects, but not in G6PD-normal subjects. Results shown with reference to primaquine 15 mg × 14 days. (**B**) Dose–response for the maximum change in hematocrit from pretreatment values up to and including day 14 following treatment start with single-dose tafenoquine 100–300 mg in G6PD-deficient heterozygous subjects, but not in G6PD-normal subjects. Results shown with reference to primaquine 15 mg × 14 days. One G6PD-heterozygous subject (subject 272) receiving primaquine withdrew consent and consequently received an incomplete dosing regimen and was only followed until day 6 (treatment day plus 5 days of follow up); this subject had no dose-limiting toxicity and a decrease in hemoglobin of–1.3 g/dL and in hematocrit of–2.8%. See Supplemental Table 2 for individual patient data.

Compared with G6PD-normal subjects, the maximum decrease in hemoglobin in G6PD-heterozygous subjects was slightly greater for tafenoquine 100 mg, but this difference became more pronounced at tafenoquine 200 mg and was evidently greater with the 300 mg dose, with dose-limiting toxicity occurring in 3/3 subjects (Figure 2). All except one case of dose-limiting toxicity had both a ≥ 2.5 g/dL decline in hemoglobin and a decrease of ≥ 7.5% in hematocrit versus pretreatment (Figure 2). Recruitment to the primaquine cohort was stopped after 3/5 G6PD-heterozygous subjects had dose-limiting toxicity. Of these three cases, all had a ≥ 2.5 g/dL decline in hemoglobin, and 2/3 also had a decrease of ≥ 7.5% in hematocrit versus pretreatment (Figure 2). In the primaquine arm, G6PD-heterozygous subjects who completed the study received 6, 9, 10, and 14 days of treatment, and the subject that withdrew consent received 6 days of treatment; all G6PD-normal subjects received 14 days of treatment. Hemolytic event frequency is summarized in Table 2. No subject had a hemoglobin value < 9.0 g/dL or a hematocrit value < 27.1% throughout the study (Supplemental Table 2).

**Table 2:** Number of G6PD-heterozygous and G6PD-normal subjects with hemolytic events following single-dose tafenoquine or primaquine 15 mg × 14 days occurring up to and including day 14 after therapy start

Event	TQ 100 mg		TQ 200 mg		TQ 300 mg		PQ 15 mg × 14 days	
	Normal (*N* = 6)	Het. (*N* = 6)	Normal (*N* = 6)	Het. (*N* = 6)	Normal (*N* = 6)	Het. (*N* = 3)	Normal (*N* = 6)	Het.‡ (*N* = 5)
Dose-limiting toxicity*	0	0	0	2	0	3	0	3
Hemoglobin decline of ≥ 2.5 g/dL	0	0	0	1	0	3	0	3
Hematocrit decrease of ≥ 7.5%	0	0	0	2	0	3	0	2
Hematologic toxicity†	3	6	5	4	0	3	6	4
Hemoglobin decline of ≥ 1.5 g/dL	2	4	2	4	0	3	0	4
Hematocrit decrease of ≥ 4.5%	2	4	3	3	0	3	1	4
Indirect bilirubin increase of > 50% from pretreatment	1	4	3	2	0	1	5	4
Haptoglobin value of ≤ 0.25 μg/dL	0	0	0	1	0	3	0	3
Reticulocytes ≥ 4%	0	4	0	3	0	2	0	3

G6PD = glucose-6-phosphate dehydrogenase; Het. = G6PD heterozygous; PQ = Primaquine; TQ = Tafenoquine.

* Hemoglobin decline of ≥ 2.5 g/dL and/or hematocrit decrease of ≥ 7.5% from pretreatment. Pretreatment levels were calculated as the mean hemoglobin or hematocrit of day −1 and day 1 (prior to drug treatment).

† Any occurrence of hemoglobin decline of ≥ 1.5 g/dL, hematocrit decrease of ≥ 4.5%, indirect bilirubin increase of > 50% from pretreatment, haptoglobin value ≤ 0.25, or reticulocytes ≥ 4%.

‡ One subject received primaquine for only 6 days.

**Figure 3.**
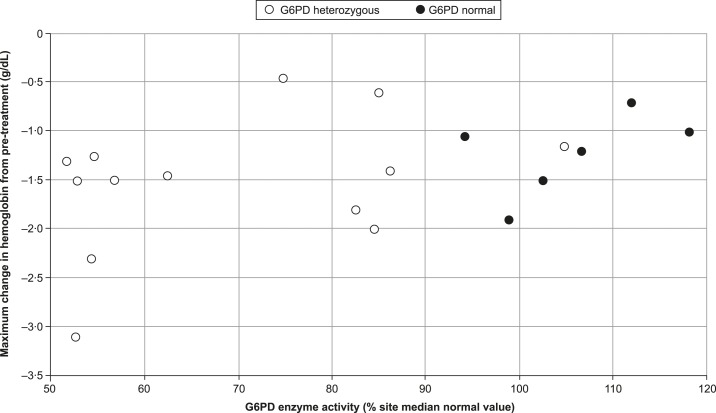
Relationship between the hemoglobin decline following single-dose tafenoquine (200 mg) and baseline glucose-6-phosphate dehydrogenase (G6PD) activity in G6PD-deficient heterozygous or G6PD-normal subjects and G6PD activity.

The hemolytic response in subjects with a wider range of enzyme activities (61 to > 80%) was explored at the tafenoquine 200 mg dose (i.e., the protocol-defined maximum tolerated dose for G6PD-heterozygous subjects with 40–60% enzyme activity). Although there were insufficient data for a formal statistical analysis, the two G6PD-heterozygous subjects who had the greatest declines in hemoglobin also had lower G6PD enzyme activities of 52.6% and 54.3% the site median value (Figure 3).

In G6PD-deficient heterozygous subjects, the time course and magnitude for the hemoglobin decline following single-dose tafenoquine 300 mg appeared similar to that of primaquine (Figure 3), despite the lower overall primaquine dose (210 mg) which was divided over 14 days. As primaquine is rapidly metabolized, this is not thought to be a drug accumulation effect.^24^

**Figure 4.**
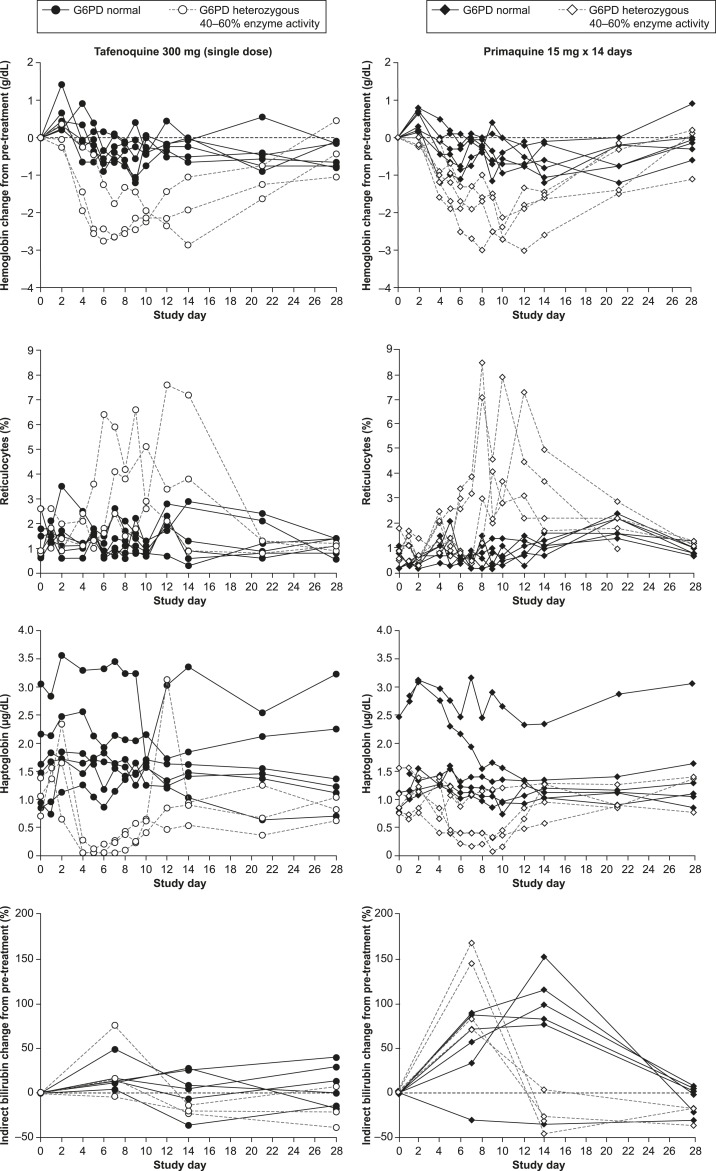
Time course of hematological parameters in glucose-6-phosphate dehydrogenase (G6PD)-normal subjects and in G6PD-deficient heterozygous subjects upon administration of single-dose tafenoquine 300 mg vs primaquine 15 mg × 14 days.

The hemoglobin nadir occurred between day 6–14 with tafenoquine 300 mg and day 8–12 for primaquine (Figure 4, Supplemental Table 2). A moderate reticulocyte response, temporally following the decline in hemoglobin, was observed consistently for G6PD-heterozygous subjects receiving tafenoquine 300 mg or primaquine (Figure 4). Reticulocytes exceeded 4% in 2/3 G6PD-heterozygous subjects in the tafenoquine 300 mg group and 3/5 in the primaquine group (Table 2).

Indicative of intravascular hemolysis, haptoglobin was rapidly depleted in G6PD-heterozygous subjects with values ≤ 0.25 μg/dL in 3/3 subjects in the tafenoquine 300 mg group and 3/5 in the primaquine group, before recovery started at around day 9 (Figure 4, Table 2).

Consistent increases in indirect bilirubin were not evident following tafenoquine 300 mg. Increases in indirect bilirubin did occur with primaquine, (20–169%), and were > 50% in 5/6 G6PD-normal and 4/4 evaluable G6PD-heterozygous subjects (Figure 4).

Normal menses are not thought to affect G6PD-associated hemolysis risk, and no effect on hemolytic potential was found in this study (data not shown).

### Methemoglobin.

**Figure 5.**
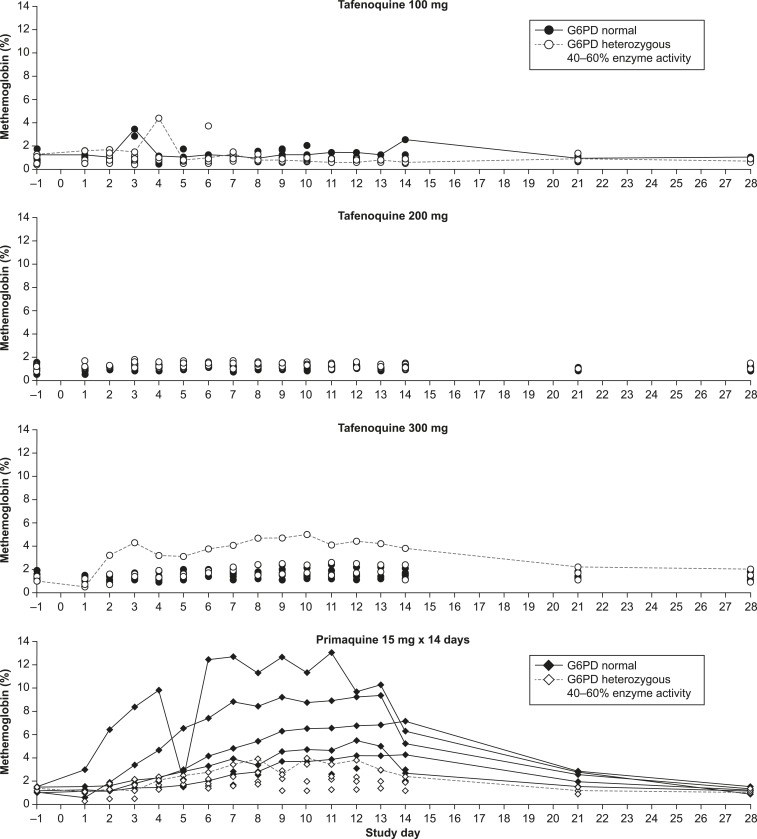
Methemoglobin values in glucose-6-phosphate dehydrogenase (G6PD)-heterozygous or G6PD-normal subjects following single-dose tafenoquine 100, 200 or 300 mg or primaquine 15 mg × 14 days. Not all profiles are drawn for reasons of clarity.

Methemoglobinemia is a known effect of 8-aminoquinolines. With tafenoquine, no subject had methemoglobin > 5.0% (Figure 5). In a recent clinical trial, no clinically important methemoglobin increases were noted with tafenoquine 300 mg in G6PD-normal patients with *P. vivax* infection.^17^

Following primaquine 15 mg × 14 days, 4/6 G6PD-normal subjects had sustained elevations in methemoglobin with maximum values between 5.5% and 13.1% (Figure 5), consistent with previous findings.^25^ In contrast, in G6PD-heterozygous subjects receiving primaquine, methemoglobin did not exceed 3.9%. In vitro experiments show primaquine dose-dependent methemoglobin accumulation in both G6PD-normal and G6PD-deficient erythrocytes.^26^

### Safety.

There were no accompanying clinical symptoms associated with hemolysis or increased methemoglobin (Supplemental Table 3). Except for the hematologic changes described earlier, there were no other clinically important changes in laboratory measures. There were no notable electrocardiogram changes.

### Pharmacokinetics.

The pharmacokinetic profile of tafenoquine in G6PD-heterozygous and G6PD-normal subjects appeared similar, as did that of primaquine (Supplemental Figure S1). There was no relationship between the severity of hemolysis and the pharmacokinetic parameters of either drug.

## DISCUSSION

This is the first study to quantify the potential for drug-induced hemolysis in G6PD-heterozygous females for any drug, including primaquine. In G6PD-heterozygous healthy female subjects with moderate G6PD enzyme activity, tafenoquine-induced hemolysis increased with increasing dose. Also, the greatest declines in hemoglobin following tafenoquine were observed in those with lower levels of G6PD activity.

An important characteristic of tafenoquine is its pharmacokinetics, whereby a single dose is curative.^17^ The long half-life of tafenoquine 300 mg, which allows single-dose therapy, did not appear to result in a higher risk of hemolytic anemia relative to that of primaquine 15 mg × 14 days (Figure 3). Of course, should hemolysis progress to more severe AHA, the primaquine course can be interrupted, whereas single-dose tafenoquine cannot be “stopped” once administered. It has been shown for the G6PD *A*– variant that continued primaquine administration leads to stabilization of hemoglobin levels, termed the “resistance phase”; a state of low-grade hemolysis resulting from higher G6PD activity in the younger red cell population following acute hemolysis.^27^ Recent data also indicate that a resistance phase can be established with primaquine for the G6PD *Viangchan* variant.^28^ Given the similarity between primaquine and tafenoquine in the time course of hematological parameters reported here, we suggest that both drugs may induce a resistance phase in *G6PD Mahidol*.

Potential differences were suggested in the hemolytic mechanism for tafenoquine and primaquine. With tafenoquine, haptoglobin decreased markedly with minimal effect on indirect bilirubin, suggesting that hemolysis was almost exclusively intravascular. With primaquine, though haptoglobin was also depleted, increases in indirect bilirubin were observed in G6PD-heterozygous and G6PD-normal subjects. Increases in indirect bilirubin are primarily associated with extravascular hemolysis.^29^ G6PD-unrelated, extravascular hemolytic mechanisms have been suggested for the primaquine metabolite 6-methoxy-8-hydroxylaminoquinoline, contributing to its overall hemolytic potential and to methemoglobin formation.^30^ In contrast, there are no detectable major metabolites of tafenoquine in human plasma or urine (detection limit 1–2 ng/mL), and tafenoquine causes less methemoglobinemia compared with primaquine.

G6PD is highly polymorphic, with different variants prevailing in different populations.^31^ All subjects included in this study were heterozygous for the *G6PD-Mahidol*^*487A*^ mutation (MIM no. 305900.005). This variant is common in southeast Asia and has been shown to reduce *P. vivax*, but not *P. falciparum*, parasite density in humans.^10^ Strictly speaking, one cannot automatically extrapolate the findings of this study to heterozygotes for other G6PD-deficient variants. However, repeating this kind of study for each G6PD polymorphic variant would be difficult, as recruitment to the study was challenging and there are nearly 200 variants, and probably more yet to be identified. It seems reasonable to assume that these results apply at least to variants with a modal enzyme activity level similar to or higher than that of *G6PD Mahidol*, for example, G6PD *A*− and G6PD *Viangchan*.^32,33^

A limitation of this study is the small number of subjects evaluated and the narrow range of enzyme activities examined. A further consideration is that these results may not be indicative of AHA severity in patients infected with malaria. Malaria is itself a hemolytic condition; one of the hematologic consequences of the parasite/host interaction is anemia. As shown for dapsone,^13^ and likely to be the case with primaquine and tafenoquine, in G6PD-deficient individuals, hemolysis and AHA result from a more complex parasite/drug/host interaction. Thus, even though G6PD-deficient patients are excluded from tafenoquine Phase IIb/III studies, these include close examination of hemolytic safety in *P. vivax* patients.^17,34^

For clinical deployment of tafenoquine, G6PD-deficient hemizygous males and homozygous females should be excluded from treatment. In most cases, the G6PD status will not be known, and it is important to have access to a point-of-care G6PD testing kit.^35^ Such kits already exist, and it is expected that a PATH-led collaborative effort will make G6PD testing even simpler, more reliable, and less expensive. The current study provides the first objective evidence that AHA risk in G6PD-heterozygous females is related to the level of G6PD enzyme activity. Consequently, a follow-up study will aim to refine further the appropriate G6PD activity cut-off level to enable access to tafenoquine treatment for the proportion of the G6PD heterozygous *P. vivax* patients that are at least risk of AHA.

Unfortunately, many drugs predictably cause oxidative hemolysis in G6PD-deficient individuals, for example, dapsone, methylene blue, ciprofloxacin, and ofloxacin.^32^ The list is extended if drugs are included that can possibly cause hemolysis in some G6PD-deficient individuals, for example, chloroquine, quinine, aspirin, paracetamol, and so on.^32^ However, risk of drug-related AHA, relative to G6PD enzyme activity, has not been quantified for any drug. The current study demonstrates a new approach to quantifying and comparing the hemolytic risk of drugs in G6PD heterozygous females with moderate G6PD activity which minimizes the risk to study participants, is technically feasible, and ethically acceptable.

In G6PD heterozygotes, the main determinant of the severity of drug-induced hemolysis will be the number of G6PD-deficient red cells. G6PD activity measured quantitatively by spectrophotometric assay in a hemolysate,^36^ is the average of the activity in G6PD-normal and G6PD-deficient red cells; a good surrogate for the proportion of G6PD-deficient red cells. Quantification of G6PD-deficient red cells can be performed using cytochemical methodologies (e.g., cyanmethemoglobin elution method,^37^ cytochemical staining^38^), and techniques have been optimized for flow cytometry.^39^

An evaluation of tafenoquine hemolytic potential in G6PD-deficiency was necessary for designing the Phase III clinical trial program. Additionally, this study has shown that G6PD enzyme levels are relevant for AHA risk, supporting the development of a point-of-care test based on G6PD phenotype. Thus, it can be envisaged that tafenoquine deployment will be possible in populations that harbor G6PD-deficient variants, whilst excluding from treatment those at most risk of AHA.

## Supplementary Material

Supplemental Table.
