# Supplementary material for: Hemolytic Potential of Tafenoquine in Female Volunteers Heterozygous for Glucose-6-Phosphate Dehydrogenase (G6PD) Deficiency (G6PD Mahidol Variant) versus G6PD-Normal Volunteers
Source: Am J Trop Med Hyg. 2017 Jul 24;97(3):702–11. doi: 10.4269/ajtmh.16-0779 (PMC5590573; doi:10.4269/ajtmh.16-0779)
Supplement: Supplementary file 1 [file tpmd160779.SD1.pdf]

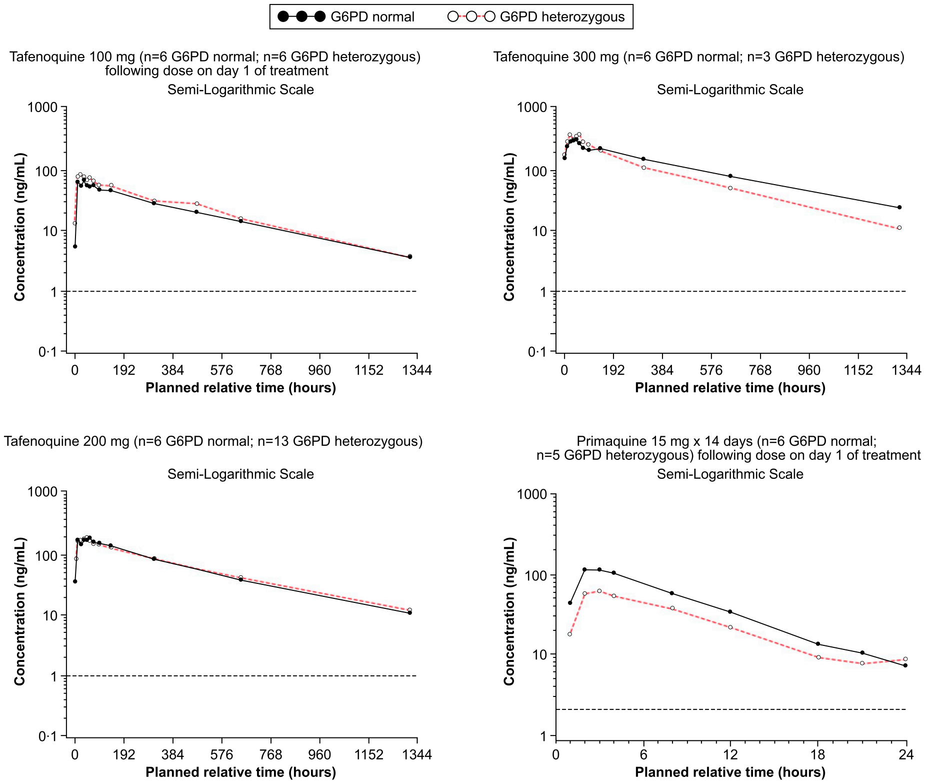

SUPPLEMENTAL FIGURE 1.

Supplemental Table 1

Normal laboratory values from the study laboratories

Test	Laboratory ID	SI result unit	Normal range lower limit in standard units	Normal range upper limit in standard units
Alanine amino transferase	A	IU/L	5.000	40.000
Alanine amino transferase	B	IU/L	0.000	40.000
Aspartate amino transferase	A	IU/L	5.000	40.000
Aspartate amino transferase	B	IU/L	0.000	37.000
Basophils	A	GI/L	0.009	0.110
Basophils	B	GI/L	0.000	0.165
Basophils (%)	A	%	0.200	1.000
Basophils (%)	B	%	0.000	1.500
Bicarbonate	A	MMOL/L	22.000	28.000
Bicarbonate	B	MMOL/L	22.000	29.000
Creatine kinase	A	IU/L	0.000	140.000
Creatine kinase	B	IU/L	0.000	167.000
Direct bilirubin	A	UMOL/L	0.000	5.985
Direct bilirubin	B	UMOL/L	0.000	8.550
Eosinophils	A	GI/L	0.040	0.319
Eosinophils	B	GI/L	0.000	0.770
Eosinophils (%)	A	%	0.900	2.900
Eosinophils (%)	B	%	0.000	7.000
G6PD	A		–	–
G6PD	B		–	–
Haptoglobin	C	G/L	0.300	2.000
Hematocrit	A	1	0.370	0.470
Hematocrit	B	1	0.370	0.520
Hemoglobin	A	G/L	120.000	160.000
Hemoglobin	B	G/L	120.000	180.000
Indirect bilirubin	A	UMOL/L	–	–
Indirect bilirubin	B	UMOL/L	–	–
Lymphocytes	A	GI/L	0.902	5.005
Lymphocytes	B	GI/L	0.760	5.280
Lymphocytes (%)	A	%	20.500	45.500
Lymphocytes (%)	B	%	19.000	48.000
Mean corpuscle hemoglobin concentration	A	G/L	320.000	360.000
Mean corpuscle hemoglobin concentration	B	G/L	310.000	350.000
Mean corpuscle volume	A	FL	81.000	99.000
Mean corpuscle volume	B	FL	80.000	99.000
Methaemoglobin (%)		%	–	–
Monocytes	A	GI/L	0.242	1.287
Monocytes	B	GI/L	0.136	0.990
Monocytes (%)	A	%	5.500	11.700
Monocytes (%)	B	%	3.400	9.000
Platelet count	A	GI/L	130.000	440.000
Platelet count	B	GI/L	150.000	440.000
Potassium	A	MMOL/L	3.700	5.300
Potassium	B	MMOL/L	3.500	5.000
Red blood cell count	A	TI/L	4.200	5.400
Red blood cell count	B	TI/L	4.200	5.400
Reticulocytes	A	TI/L	0.025	0.140
Reticulocytes	B	TI/L	0.021	0.108
Reticulocytes (%/ratio)	A	1	0.006	0.026
Reticulocytes (%/ratio)	B	1	0.005	0.020
Segmented neutrophils	A	GI/L	1.892	7.150
Segmented neutrophils	B	GI/L	1.600	8.140
Sodium	A	MMOL/L	135.000	148.000
Sodium	B	MMOL/L	135.000	145.000
Total bilirubin	A	UMOL/L	3.420	17.100
Total bilirubin	B	UMOL/L	5.130	20.520
Total neutrophils (%)	A	%	43.000	65.000
Total neutrophils (%)	B	%	40.000	74.000
Urea/BUN	A	MMOL/L	1.785	8.211
Urea/BUN	B	MMOL/L	2.499	7.140
White blood cell count	A	GI/L	4.400	11.000
White blood cell count	B	GI/L	4.000	11.000

A = Clinical Pathology Laboratory, Mae Sot General Hospital, 175/16 Sripanich Road, Mae Sot 63110, Thailand; B = Department of Clinical Pathology, Siriraj Hospital, Bangkok, Thailand; C = Quest Heston, UK; G6PD = glucose-6-phosphate dehydrogenase.

Supplemental Table 2

Individual subject data for G6PD enzyme activity, hemoglobin, and hematocrit at pretreatment, and nadir values and maximum absolute decline in hemoglobin and hematocrit from pretreatment up to and including day 14 following administration of single-dose tafenoquine or primaquine 15 mg × 14 days

				Hemoglobin, g/dL			Hematocrit, %		
Treatment	G6PD status	Patient	G6PD enzyme activity, % site median	Pretreatment	Nadir	Maximum decline	Pretreatment	Nadir	Maximum decline
Tafenoquine 100 mg	Normal	6	123.92	12.5	10.9	−1.6	37.9	33.2	−4.7
		13	56.07	12.4	10.9	−1.5	38.1	34.1	−4.0
		17	83.36	12.5	11.3	−1.2	38.1	35.5	−2.6
		19	38.47	12.2	11.6	−0.6	36.9	34.7	−2.2
		24	183.71	12.2	11.2	−1.0	35.7	32.4	−3.3
		26	201.91	14.3	12.4	−1.9	42.4	36.9	−5.5
	Heterozygous	32	18.98	12.5	10.1	−2.4	37.8	31.4	−6.4
		38	49.91	12.9	11.1	−1.8	39.1	33.3	−5.8
		39	37.00	12.3	11.4	−0.9	36.4	34.8	−1.6
		41	39.69	13.4	12.3	−1.1	40.4	37.6	−2.8
		113	18.89	12.8	10.8	−2.0	39.4	32.6	−6.8
		121	23.74	13.2	11.1	−2.1	39.0	33.3	−5.7
Tafenoquine 200 mg	Normal	130	106.67	14.6	13.4	−1.2	43.4	38.3	−5.1
		131	111.96	12.9	12.2	−0.7	38.0	35.6	−2.4
		132	102.51	14.1	12.6	−1.5	42.1	36.8	−5.3
		133	98.87	13.4	11.5	−1.9	39.7	33.6	−6.1
		142	118.11	12.7	11.7	−1.0	37.1	33.7	−3.4
		148	94.19	12.3	11.2	−1.1	36.3	33.8	−2.5
	Heterozygous	140	52.60	12.5	9.4	−3.1	38.3	27.8	−10.5
		152	56.76	12.5	11.0	−1.5	37.0	32.5	−4.5
		179	52.77	12.8	11.3	−1.5	42.5	35.2	−7.3
		194	54.59	12.7	11.4	−1.3	38.6	34.5	−4.1
		202	51.65	12.6	11.3	−1.3	38.0	34.4	−3.6
		210	54.33	13.1	10.8	−2.3	38.6	31.0	−7.6
Tafenoquine 300 mg	Normal	225	96.01	15.0	14.6	−0.4	43.8	41.8	−2.0
		227	108.32	13.6	12.4	−1.2	40.4	36.0	−4.4
		231	162.91	12.8	12.1	−0.7	39.6	36.5	−3.1
		232	135.01	13.1	12.4	−0.7	39.2	36.5	−2.7
		233	110.23	12.8	11.7	−1.1	38.7	34.3	−4.4
		243	102.17	12.2	11.6	−0.6	37.0	35.3	−1.7
	Heterozygous	217	56.93	12.3	9.3	−3.0	35.9	27.1	−8.8
		220	54.07	12.7	9.9	−2.8	37.7	29.2	−8.5
		245	40.99	12.4	9.7	−2.7	36.9	28.6	−8.3
Primaquine 15 mg × 14 days	Normal	248	101.99	13.0	12.3	−0.7	37.6	34.7	−2.9
		252	106.24	12.5	11.3	−1.2	36.8	31.8	−5.0
		253	118.37	12.4	11.6	−0.8	35.9	33.5	−2.4
		256	98.35	12.2	12.0	−0.2	35.6	34.9	−0.7
		258	113.26	13.3	12.1	−1.2	38.4	35.2	−3.2
		259	110.05	12.2	11.1	−1.1	36.2	32.6	−3.6
	Heterozygous	272*†	51.91	13.0	11.7	−1.3	38.3	35.5	−2.8
		276*	44.02	12.9	10.4	−2.5	36.4	29.6	−6.8
		282*	57.80	13.7	10.7	−3.0	40.8	30.2	−10.6
		299	47.75	12.6	10.5	−2.1	38.7	32.0	−6.7
		330*	43.59	12.0	9.0	−3.0	37.1	27.8	−9.3
Tafenoquine 200 mg	Heterozygous A	284	74.78	14.1	13.6	−0.5	40.6	39.9	−0.7
		311	62.39	12.1	10.6	−1.5	37.3	32.3	−5.0
	Heterozygous B	290	82.50	12.3	10.5	−1.8	36.5	30.2	−6.3
		292	86.22	12.5	11.1	−1.4	37.3	32.2	−5.1
		294	85.01	12.2	11.6	−0.6	36.7	35.2	−1.5
		301	84.49	13.4	11.4	−2.0	38.8	32.7	−6.1
		308	104.77	12.6	11.4	−1.2	38.1	35.0	−3.1

Heterozygous = G6PD heterozygous 40–60% enzyme activity of site median normal value (19–50% for tafenoquine 100 mg); Heterozygous A = G6PD heterozygous 61–80% enzyme activity of site median normal value; Heterozygous B = G6PD heterozygous > 80% enzyme activity of site median normal value. Note that pretreatment values for hemoglobin and hematocrit were the mean of day −1 and predose day 1 values.

* Four subjects received an incomplete primaquine dose: 6 days for subject 272; 9 days for subject 276; 10 days for subject 282; and 6 days for subject 330.

† All subjects in the study completed follow up to day 55, except for subject 272 who withdrew from the study on day 5 (treatment start was on day 0 plus 5 days of follow up).

Supplemental Table 3

Treatment-emergent adverse events following administration of single-dose tafenoquine or primaquine 15 mg × 14 days

Event, number of patients	Tafenoquine 100 mg		Tafenoquine 200 mg		Tafenoquine 300 mg		Primaquine 15 mg × 14 days	
	Normal (*N* = 6)	Het. (*N* = 6)	Normal (*N* = 6)	Het. (*N* = 13)	Normal (*N* = 6)	Het. (*N* = 3)	Normal (*N* = 6)	Het. (*N* = 5)
Any event	6	4	0	2	0	3	0	3
Hemoglobin decreased	3	1	0	0	0	3	0	2
Hematocrit decreased	0	0	0	1	0	0	0	1
Alanine aminotransferase increased	0	0	0	1	0	0	0	0
Aspartate aminotransferase increased	0	0	0	1	0	0	0	0
Dizziness	0	1	0	1	0	0	0	0
Headache	1	1	0	0	0	0	0	0
Nasopharyngitis	2	1	0	0	0	0	0	0
Nausea	1	0	0	0	0	0	0	0
Pyrexia	1	0	0	0	0	0	0	0
Myalgia	0	1	0	0	0	0	0	0
Rhinorrhea	1	0	0	0	0	0	0	0

Het. = G6PD heterozygous.
